# Beneficiary and Local Stakeholder Participation in Community-Based Nutrition Interventions

**DOI:** 10.1093/cdn/nzac131

**Published:** 2022-08-29

**Authors:** Rebecca C Robert, Brittany L Feijoo

**Affiliations:** Conway School of Nursing, The Catholic University of America, Washington, DC, USA; Conway School of Nursing, The Catholic University of America, Washington, DC, USA

**Keywords:** participatory research, community participation, nutrition-sensitive agriculture interventions, nutrition-specific interventions, Africa, stakeholders, beneficiaries, maternal child nutrition

## Abstract

Beneficiary and local stakeholder participation is an essential element to the success of community-based nutrition interventions. We sought to define active participation and review the available evidence on beneficiary and local stakeholder participation in community-based nutrition interventions in Africa. From reviewing the literature, we provide a reflective assessment on the process and findings. Participation falls on a continuum of community involvement from passive (no real involvement) to empowerment and community ownership (full active involvement). However, we found a clear gap in the research on defining active participation and identifying what constitutes active participation on behalf of beneficiaries and local stakeholders. However, progress was found; evidence included the use of participatory methods to engage beneficiaries and local stakeholders in the assessment and design phase. Beneficiary and local stakeholder participation in delivering interventions has moved forward with quantitative measures from process evaluation and implementation science. Research has started on the extent of beneficiary engagement (as recipients) and connecting this to outcomes. Evaluation has benefited from qualitative inquiry with insights from participants on engagement itself, and the barriers and facilitators to engagement. Yet questions remain in each study phase around defining and quantifying active participation and in understanding the personal, social, and motivational elements of active participation. We offer a simple framework to stimulate thought and commitment to research on participation in community-based nutrition interventions.

Sustainable Development Goal #2 addresses the direct importance of nutrition, stating to “End hunger, achieve food security and improved nutrition, and promote sustainable agriculture” (1). Moreover, it is thought that improved nutrition could impact ≥12 of the 17 sustainable development goals, signifying its extensive potential impact (1). Addressing nutrition across various sectors and contexts is key to accomplishing these international goals. This recognition has led to investments in nutrition-specific and nutrition-sensitive agriculture interventions that focus on improving community-based nutrition by addressing the direct and underlying causes of malnutrition (2, 3). In 2021, the *Lancet* published an updated framework on nutrition actions and updates on the evidence for nutrition-specific and nutrition-sensitive interventions to improve maternal, child, and adolescent nutrition (4). We assert that active participation of beneficiaries and local stakeholders in community-based nutrition interventions is an essential element in their success—deriving maximum benefits for improved nutrition. The WHO defines participation as a “key driver of health equity” leading to positive health outcomes and well-being (5). But how is active participation defined? What is the available evidence on beneficiary and local stakeholder participation in community-based nutrition interventions? Moreover, what are the existing gaps and how can this inform future research on active participation and lead to successful outcomes from community-based nutrition interventions?

We began by searching for a general definition of participation in community-based interventions, which led us to global health literature on primary care. There we found authors citing the lack of a precise definition and instead defining participation by a continuum of community involvement from passive (no real involvement) to empowerment and community ownership (full active involvement) (4, 6–11). On this continuum, the ends were fairly well defined, yet the middle areas, which detail stages of increasingly more active participation (e.g., community compliance, consultation, collaboration, and co-learning), were decidedly “gray.” This left us without clear guidance as to how to define active participation by beneficiaries (the direct recipients of interventions) or local stakeholders (indirect recipients of interventions, such as family). Moreover, community participation also refers commonly to the delivery of interventions by local stakeholders (e.g., community health workers), which adds to the complexity of what to label as active participation. Beneficiaries and local stakeholders might actively participate in other phases of community interventions (e.g., design or evaluation), also meriting consideration.

The continuum of beneficiary and local stakeholder participation in community-based interventions makes clear the importance of participation as a process toward community empowerment—the social transformation of “those without power gaining information, skills, and confidence and thus control over decisions about their lives, and can take place on an individual, organizational, and community level” (11). This reflects the Alma Ata Declaration of Primary Health Care, stating, “The people have the right and duty to participate individually and collectively in the planning and implementation of their health care,” and is consistent with the goal of self-reliance and health equity (5, 12).

We turned to the literature to seek clarity on the available evidence of community participation by conducting a thorough search and review of the literature on community-based nutrition interventions in Africa. We developed a search term syntax (e.g., nutrition, community, participation, engagement, stakeholders, intervention, Africa) to capture a wide range of available studies in several databases and screened >1600 articles from the past 15 y. We selected >100 articles on community-based nutrition interventions—either nutrition-specific or nutrition-sensitive agriculture interventions—including quantitative, qualitative, and mixed methods studies—and categorized them by 3 study phases: assessment and design, implementation, or evaluation (with some studies in overlapping categories).

In the process of reviewing the studies, we found the concept of participation comprised a wide-ranging, diverse, and sometimes overlapping concept. It referred to: *1*) beneficiary and local stakeholder engagement in identifying problems, prioritizing solutions, and designing context-specific interventions; *2*) involvement in the implementation of interventions—that is, actually delivering interventions (e.g., by community health workers) or mobilizing beneficiaries to engage with interventions; and/or *3*) extent of engagement in terms of receiving interventions, that is, the degree to which beneficiaries and local stakeholders are exposed to, engage with, initially use, or uptake interventions. Participation also included beneficiary and local stakeholder involvement in evaluation through *4*) collecting data to monitor community metrics; *5*) providing feedback to researchers on interventions (e.g., on satisfaction) and offering perspectives on the barriers and facilitators to engagement and on how to improve interventions. Finally, participation referred to: *6*) engaging in the process towards community empowerment, or more commonly women's empowerment, a secondary objective of many nutrition-sensitive agricultural interventions.

The lack of clarity and consensus on what active participation is comprised of and the rather implausible task of organizing and synthesizing the diverse scope of studies reviewed into a cogent framework shifted our focus. Specifically, we adapted our intended state-of-the-art review to a reflective assessment of the literature on participation in community-based nutrition interventions. This provided us the opportunity to step back and view the gaps and progress made thus far, offer direction for further studies, and provide a framework to inspire and guide future research on active participation in community-based nutrition interventions.

Most of the studies we reviewed focused either on maternal, infant, and child nutrition or infant and child nutrition specifically. They included nutrition-specific and nutrition sensitive agriculture interventions. A mix of quantitative and mixed methods studies addressed implementation and evaluation most commonly, whereas qualitative studies dominated the assessment and design phase of community-based nutrition interventions. Several studies incorporated known participatory approaches throughout (e.g., participatory-based community research, participatory action research). Studies were reviewed from many African countries including Ghana, Kenya, Malawi, Ethiopia, and Burkina Faso among others, offering a diversity of community contexts.

In the assessment and design phase of community-based nutrition interventions, the use of known participatory methods easily identifies active participation. For example, participatory video (13), photovoice (14), and trials for improved practices—a participatory method engaging beneficiaries to test the feasibility and acceptability of new practices and offer recommendations (15–19). Other studies include participatory workshops in which researchers and local stakeholders discuss and prioritize solutions (20, 21).

However, in some studies, active participation might not be as apparent. For example, focus group discussions as part of formative research might ask the group to identify problems and offer solutions (22–26). Likewise, in focused ethnographic studies, researchers might engage participants in interviews and cognitive mapping exercises (e.g., pile sorts, free listing) to gain deeper understanding of local context and culture, but might not ask for specific solutions (27–29). Are these sufficient to be considered active participation? Does testing or adapting materials with participants in a focus group discussion elevate to participation (30, 31)? These represent the gray or vague areas of what to consider active participation. Regardless, no one would deny the importance and contribution of any of these studies. In other instances, the stage of research could require a deeper understanding of the problem through engaging local stakeholders with in-depth interviews or observations, which again lacks the clear description of active participation (32, 33).

Regarding the implementation phase, implementation science has propelled the field forward with its use of context-specific program impact pathways to convey how nutrition-specific and nutrition-sensitive interventions intend to achieve their outcomes (34). Yet, the very nature of interventions differs, with some inherently more participatory focused. Program impact pathways assist in understanding who is involved in delivery (e.g., participation of local stakeholders such as community health workers) and what they are expected to deliver. Pathways also include the expected participation/engagement by beneficiaries. The detail and complexity within these project-specific pathways varies as does the associated metrics to measure them.

In particular, participation in the delivery of interventions has advanced with the use of standard (more or less) process indicators (e.g., dose delivered, fidelity) applied to the context-specific activities of community-based nutrition interventions (35–40). The number and detail vary by project. What level of participation indicates active participation? For example, is completing 50% compared with 90% of planned activities considered differently in terms of active participation? Should incentivized compared with volunteer local stakeholders be held to the same expectations for active participation? What threshold level of active participation is actually needed to engage beneficiaries and produce change? Beyond objective measures, what do local stakeholders gain from their involvement as active participants in delivering interventions? Qualitative data might better capture additional underlying concepts of what active participation entails (41–43).

On the beneficiary side of participation, the extent of engagement with an intervention is generally measured by the process indicator “dose received.” This is defined as exposure, engagement with, initial use, or uptake of the intervention (35). However, the extent of participatory effort involved in exposure to an intervention (e.g., received a community health worker home visit) can be quite different than taking an action of initial use (e.g., planting vines). Studies use a variety of contextualized indicators of dose received to measure beneficiary participation (44, 45). Researchers can create scales or scores from several indicators of beneficiary participation or engagement to provide for a more complete assessment (20, 46–49). These vary by project, including the methods for creating them and what they represent (e.g., participation level, participation effort, intensity of exposure), making comparisons of active participation across studies challenging. However, including a variable in statistical models to represent different levels of beneficiary participation provides insight on the influence of participation on project outcomes. Questions remain about the best mix of items to capture active participation and how the project context influences this. Qualitative inquiry is also employed to explore better understanding of beneficiary participation (42, 50–52). This type of inquiry is critical to understand the motivational factors underpinning active beneficiary engagement and the personal changes that occur from participation.

The evaluation stage of a study can also actively engage beneficiaries or local stakeholders, seeking opinions on the intervention from their involvement, such as level of satisfaction or perceived value. These measures can be captured through quantitative measures or qualitative exploration but often blend or overlap with what is considered implementation data (44, 50, 53–55). Participants’ perceptions on the barriers and facilitators to participation and their perspectives on what drives successful engagement provide valuable feedback for researchers (38, 43, 56, 57). Intervention projects that build in iterative processes for change can benefit from engaging beneficiaries and local stakeholders in making recommendations. But, how do we determine the level of active beneficiary and local stakeholder participation from these varying methods of engagement in evaluation?

Examples of nutrition-sensitive agriculture studies exist that have adopted a community-based participatory research approach—defined as “action oriented and [equitably] community-partnered” (21, 58–61). Such studies aim for researchers, local stakeholders, and beneficiaries to partner in defining, implementing, and evaluating interventions to improve nutrition outcomes and foster the processes of community empowerment. In nutrition-sensitive agriculture interventions, women's empowerment can underlie or form a secondary objective.

We began this article by asking the question, what is the available evidence on beneficiary and local stakeholder participation in community-based nutrition interventions in Africa? What we found was a clear gap in the research on defining active participation and identifying what constitutes active participation on behalf of beneficiaries and local stakeholders. This held true in every study phase of community-based nutrition interventions. Increased and intentional inclusion of active participation throughout study phases will advance our understanding of what, when, and how it can contribute to community-based nutrition interventions and better nutrition outcomes.

We see progress, particularly in the use of participatory methods to engage beneficiaries and local stakeholders in the assessment and design phase. Beneficiary and local stakeholder participation in delivering interventions has moved forward with quantitative measures from process evaluation and implementation science. Research has started on the extent of beneficiary engagement (as recipients) and connecting this to outcomes. Evaluation has benefited from qualitative inquiry with insights of participants on engagement itself, and the barriers and facilitators to engagement.

More work remains to define and quantify active participation, but also in terms of the personal, social, and motivational elements that individuals and communities may gain from engagement and active participation. The focus on beneficiary and local stakeholder participation is welcome in this supplemental issue and highlights how we can advance research in this area. Further, we recommend focused literature reviews of current research on active participation, for example, of a particular study phase or defined piece of a study phase (e.g., extent of beneficiary engagement), or reviews of similar large-scale projects (e.g., nutrition-sensitive agricultural interventions) in similar or different contexts. Participation is complex and research is needed to unpack this concept, and answer the many questions posed in this article.

We offer a simple framework to stimulate thought and commitment to research on participation in community-based nutrition interventions. Community empowerment sits at the center to remind researchers of how participation in any study phase(s) or context can facilitate movement toward community empowerment alongside nutrition goals (**Figure 1**). Questions to consider within each intervention phase include *who* will participate, the *purpose* of their participation, the *process or methods* of participation/engagement, the *description or measurement* of participation, and finally the *outcomes* of participation (8).

**FIGURE 1:**
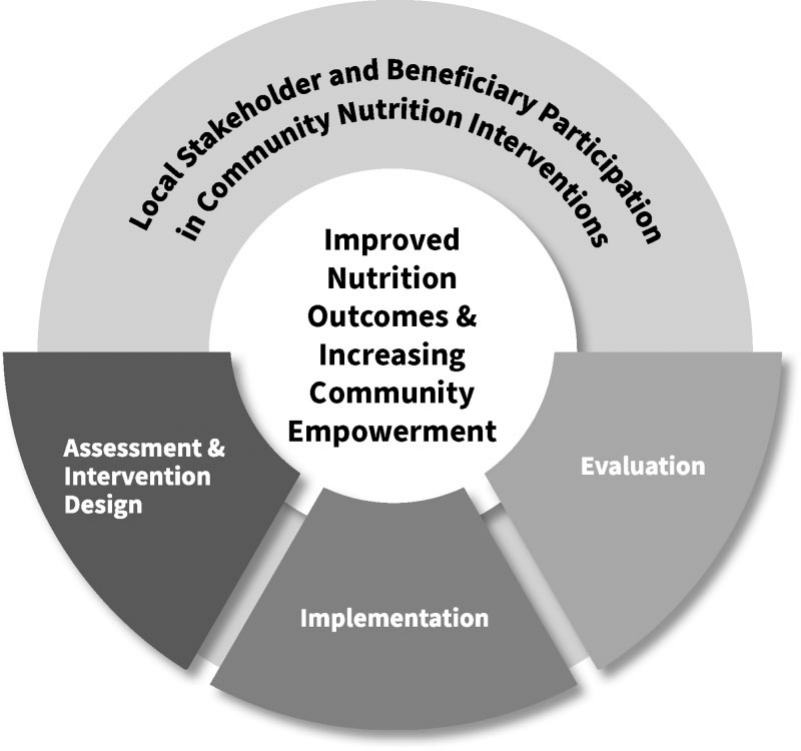
Framework for engaging beneficiary and local stakeholder participation.

As research begins to define the essential elements of active participation, community-based nutrition interventions can benefit—unlocking their potential to improve nutrition globally.

## Data Availability

Data described in the manuscript, code book, and analytic code will not be made available because this manuscript does not contain original data.

## Notes

The authors reported no external funding for the preparation of this manuscript.

Author disclosures: The authors report no conflicts of interest.
